# On the Means of Detecting Arsenic in Cases of Poisoning, &c.

**Published:** 1842-01

**Authors:** 


### Art. X.

1. *Rapport sur les Moyens de constater la présence de l'Arsenic dans les Empoisonnemens, &c.* Par M. Orfila.—*Paris*, 1841. 8vo, pp. 53.

*Report on the Means of detecting Arsenic in Cases of Poisoning, &c.* By M. Orfila.—*Paris*, 1841.

2. *De l'Arsenic, suivi d'une Instruction propre à servir de Guide aux Experts dans les cas d'Empoisonnement, &c.* Par MM. Danger et Flandin.—*Paris*, 1841. 8vo, pp. 301.

*On Arsenic, with Directions for the Investigation of Cases of Poisoning, &c.* By MM. Danger and Flandin.—*Paris*, 1841.

3. *Mémoire sur plusieurs Affaires d'Empoisonnement par l'Arsenic.* (*Mém. de l'Acad. Royale de Médecine*, Tome IX.) Par M. Orfila.—*Paris*, 1841.

*Memoir on several Trials for poisoning by Arsenic.* (*From the Memoirs of the Royal Academy of Medicine*, Vol. IX.) By M. Orfila.—*Paris*. 1841.

4. *Recherches Médico-légales et Thérapeutiques sur l'Empoisonnement par l'Acide Arsénieux, &c.* Recueillies et rédigées par le Docteur Beaufort.—*Paris*, 1842. 8vo, pp. 192.

*Medico-legal and Therapeutical Researches on Poisoning by Arsenious Acid.* By Dr. Beaufort.—*Paris*, 1842.

Since the occurrence of the "procès Laffarge," there has arisen among French medical jurists and chemists a violent controversy on the value of Marsh's test in cases of arsenical poisoning. We are sorry to perceive that this has degenerated into coarse personality, and that some of the disputants, instead of showing a zeal to develope the truth, have occupied themselves in attacking each other's opinions. Science, however, has derived some benefit from the discussions that have taken place at the Royal Academies of Medicine and Sciences. Committees of able and unbiassed men have been appointed to report on the relative value of the many processes suggested for the detection of minute traces of arsenic, and the results of their labours we have prefixed to this article.

One half of the volume of Messrs. Danger and Flandin is made up of the stormy discussions at the Academy. These matters we shall avoid: our object being to show simply what progress has been made in this important branch of toxicology.

In our Number for January last (No. XXI.) we gave a lengthened notice of the Memoirs on Poisoning, published by M. Orfila; and we there threw out certain doubts on the correctness of some of his views on the subject of arsenic. Orfila's experiments have been since repeated before the committee of the academy; and it is satisfactory to us as reviewers, as well as to all those interested in medico-legal researches, to find that the report now made by the committee fully corroborates the opinions which we then expressed.

Since the notice above referred to Orfila's processes for detecting arsenic have undergone some important modifications. In the use of Marsh's apparatus he relied chiefly upon certain chemical characters possessed by the metallic stains. When the quantity of poison is small, as must always be the case in delicate analyses where the usual tests fail, it is scarcely possible to determine these characters in the manner recommended by Orfila; hence a doubt was fairly raised on the safety of this method in cases requiring judicial investigation. MM. Danger and Flandin have fully appreciated the weight of this objection, and have endeavoured to remove it by suggesting a simple process for transforming the poison into arsenious acid, and then examining the product in the ordinary way. This is undoubtedly preferable to any analysis of stains; and it has the advantage of removing every possible objection to the use of Marsh's test. We shall commence with an examination of their work.

In giving an historical account of the processes used for detecting arsenic, the authors ascribe the merit of the discovery of the reduction of arsenic by hydrogen to their countryman Sérullas, (p. 3,) Mr. Marsh is represented to have borrowed the idea from Sérullas. We consider this decidedly unjust; no one ever heard of Sérullas' experiments on the properties of arsenuretted hydrogen until Mr. Marsh's discovery was announced; and all candid persons must agree that he alone is entitled to the merit of being the original discoverer. The advocates of Sérullas are claiming much more for him in this matter than we believe he would have claimed for himself.

MM. Danger and Flandin appear to have first directed their enquiries to the alleged existence of arsenic, as a normal constituent of certain parts of the human body. They were surprised at finding, from repeated trials, that not only did different processes give dissimilar results, but that the same process did not always give identical results. These differences were due to apparently trivial circumstances connected with the mode of operation. The plan recommended by Orfila of carbonizing the organic matter by means of nitric acid, they found to be exposed to serious defects. It is unnecessary to refer to the many trials made by them before their process was completed; but it may suffice to state at present that they could obtain no traces of arsenic from the solid, liquid, or volatile products resulting from the destructive distillation of large portions of the human body. They procured, however, stains on porcelain by the use of Marsh's apparatus, so closely resembling those of arsenic in physical and chemical characters, as to be easily confounded with them. "Thus the flame resembled that of arsenuretted hydrogen in colour and odour; the metallic stains were easily volatilized by directing on them the point of flame, they were soluble in nitric acid, and the solution thus obtained was precipitated yellow by sulphuretted hydrogen gas, and of a brick-red colour by nitrate of silver." (p. 11.) These stains were due to a combination of sulphite and phosphite of ammonia, with some volatile animal matter; and the experimentalists state that they succeeded in synthetically forming the substance from which they could be produced.*

On discovering this fact, they naturally doubted whether arsenic had not been erroneously pronounced to be a normal constituent of the human body; and the only way to remove this doubt consisted in entirely destroying every trace of organic matter in a close vessel, and submitting the pure saline residue to analysis. A somewhat complicated apparatus

* It is stated (p. 10,) that a mixture of 88 parts of ammonia, 10 parts of phosphite (?) of ammonia, and 2 parts of essential oil of turpentine, will, when introduced into the apparatus, yield stains resembling those of arsenic.

was employed for this purpose, and the result was as we have stated, no arsenic whatever could be detected in the organs of the body. The bones and the soft parts gave equally negative results, and there could be no doubt that the stains, pronounced to have been arsenical, were really due to products formed during the decomposition of the animal matter. The soil of different cemeteries around Paris gave stains with Marsh's apparatus, as also flour and bread; but in all these cases the stains were similar to those above mentioned, and were not of an arsenical nature.

These results were of the highest medico-legal importance; and the authors having thus disproved the existence of arsenic as a normal constituent of the body, next proceeded to experiment on animals which had been intentionally poisoned by that substance. They found that when gradually increasing doses of arsenic had been given to dogs without any apparent suffering being produced in the animal, the solid excrements alone yielded traces of the poison. None was discovered in the urine, either during life or after death; but when death was the direct result of a speedy effect of the poison, the viscera and the muscles gave evident indications of the presence of arsenic in them. The authors are at issue with Orfila as to whether animals destroyed by the acute form of arsenical poisoning micturate or not! They contend that no urine is passed, and that after death the bladder is found empty. The committee have not thought it beneath their dignity to enter into this very unimportant part of the controversy, and to make a report upon what must be really an accidental condition.

By far the most important part of the work is the process recommended for detecting arsenic in the tissues of the human organs, where the ordinary means of analysis entirely fail. It is undoubtedly true that, in numerous cases where arsenic has destroyed life, none of the poison can be detected in the cavities of the viscera; a circumstance which, although easily explicable on pathological principles, generally excites a doubt of the real cause of death in the minds of non-professional persons. Courts of justice unwillingly convict where the poison is not found in the body, and in many instances, as where a body has been long interred, a conviction would be absolutely impossible unless the poison were discovered. It is necessary to remember in these enquiries that the facility with which arsenic is detected in the dead is to be ascribed to the unnecessarily large quantity of poison which is commonly exhibited. Ordinary chemical analysis is directed to the surplus quantity left in the stomach or intestines, in other words to that portion of the poison which has not destroyed life. Copious diarrhœa or vomiting, the free exhibition of diluents, or where life is protracted, the power of absorption may entirely remove the poison from the viscera. In all these cases, the most important branch of medical evidence is lost, and should chemical evidence be deemed essential by the court the prisoner will owe his escape to the violence of the symptoms under which his victim laboured, or to the kind of treatment adopted! The credit is due to Orfila for having first proposed in these cases to seek for that portion of the poison actually absorbed into the system, which has probably caused death. He has shown that arsenic may be detected in the blood, soft organs, and muscles; and although, as we have stated elsewhere (No. XXI.), he has relied too implicitly upon the physical characters of the stains produced in the use of Marsh's apparatus—or what amounts to the same, he has trusted to chemical characters uncertain of application in such minute quantities, yet medical jurists owe much to him for pointing out the means of obtaining evidence in cases which have been hitherto abandoned as hopeless. Our authors, in following the steps of Orfila, have, to a certain extent, improved upon his process. So long as stains were considered to furnish evidence of the presence of arsenic, it was natural that a doubt should be entertained of the soundness of the opinion expressed, especially when the quantity of the supposed poison was infinitesimal, and the apparatus producing it was new and, comparatively speaking, untried. Now, however, that the poison may not only be extracted from the organs of the person poisoned, but by the use of their apparatus actually reproduced, free from any trace of organic matter in its original state of arsenious acid, so that every known test for arsenic may be applied to it, no one can hesitate to admit that a considerable step has been made in medico-legal analysis; and that we have at length attained to the same certainty of evidence with regard to the absorbed poison, which has hitherto been confined to that portion only found loosely dispersed in the stomach and intestines.

As the process of MM. Danger and Flandin has only just appeared before the public, we shall briefly describe it to our readers. It consists of two parts: 1st, the carbonization of the organic tissues, so as not to destroy the poison; and 2d, the transformation of the arsenical compound produced into arsenious acid.

The separation of organic matter in a liquid containing arsenic by the use of chemical reagents has been entirely abandoned. The only safe principle on which we can proceed is so to decompose the organic matter, as to render it perfectly insoluble in water, and at the same time while we prevent the loss of arsenic to render it easy of extraction in the state of salt, by water. These facts premised, the following description of the process will be easily understood:

"In order to decompose the animal matters, we digest them in a porcelain capsule with strong sulphuric acid, in the proportion of from $\frac{1}{6}$ to $\frac{1}{2}$ their weight, according to the quantity of fatty matter present. [The animal substance, which in an experimental trial may weigh about an ounce, is previously cut into very small pieces.] The mixture is then gradually heated to between 600° and 700° (360° c.) Carbonization takes place rapidly without any frothing, and if a sufficient quantity of sulphuric acid has been employed, the carbon resulting is dry and pulverulent, and by digestion in water a solution containing traces of arsenic might be procured. Should the carbonaceous residue be unctuous and greasy, the vessel may be withdrawn, and when cool more sulphuric acid should be added, and the mixture again evaporated to dryness. According to our experiments there is no compound of arsenic that gives a more delicate reaction with Marsh's apparatus than arsenic acid, which besides, being a deliquescent substance, can easily be separated from the insoluble matter by a very small portion of water: hence, after having thoroughly dried the carbonaceous residue, we treat it with a mixture of three parts of nitric and one part of muriatic acid, adding just enough to moisten perfectly the pulverized carbon. . . . . . When the mass has been heated to dryness, the residuary carbon is digested in a small quantity of water, and the solution filtered. The liquid thus obtained, contains so little animal matter, that it is perfectly limpid and colourless, and when introduced into Marsh's apparatus it never yields any froth." (p. 24.)

The carbonaceous residue, it is remarked in another place (p. 38), should be treated several times with water to ensure the solution of the whole of the arsenic acid, and these different solutions may be then mixed and concentrated by evaporation.

The report of the committee on this process gives some additional particulars which may be here mentioned:

"The exact quantity of sulphuric acid which it is necessary to employ cannot be determined à priori: it depends on the state of the animal substance, the more fresh and humid this is, the larger the quantity of acid required: thus when blood is under examination, the proportion of acid must be at least one half. The capsule should be equally heated: the animal matter soon liquefies and dissolves in the acid, forming a dark unctuous mass. It is to be continually stirred with a glass rod, by which vapours of sulphuric and sulphurous acids are disengaged. In about forty minutes the operation is finished, the substance being converted into a friable charcoal." (Report, p. 25.)

When cool it is reduced to a fine powder, and then treated with nitro-muriatic acid in the proportions above described. This converts the arsenic to the state of arsenic acid, which is easily dissolved out by boiling water. Such then is the first part of the process. It is of course necessary that the sulphuric acid employed should be absolutely pure and entirely free from every trace of arsenic. It seems to us that a very high temperature is employed, and that a lower heat might answer, while there would be less risk of volatilizing any of the arsenic. The authors admit that the quantity of arsenic lost amounts to about one tenth; while by Orfila's process with nitric acid, no less than eight tenths were lost. The committee report very favorably on this mode of carbonization, considering it to be simple, convenient, and rapid in its execution: while they prefer it to the process by nitric acid, they think it inferior to that by nitrate of potash, the grounds for which we propose to examine hereafter.

Thus then the poison absorbed into the body is brought to the state of arsenic acid, and there is no necessity for employing Marsh's apparatus for determining the real nature of this compound. There are many tests, including that of reduction by means of flux (for which purpose we prefer the calcined acetate of soda), which will show that we have really an arsenical compound under examination; and these tests must convince the most sceptical, that medical evidence is not strained in such cases, or based upon doubtful experiments on infinitesimal quantities.

MM. Danger and Flandin prefer transforming this compound into the state of arsenious acid; and this they accomplish easily, by adopting a very ingenious condensing apparatus to Marsh's tube, so that the arsenuretted hydrogen is continually burnt with a low flame, and the arsenic is obtained partly in the solid state and partly in the form of a strong aqueous solution. This apparatus has the advantage of being simple and inexpensive; it may be safely used by those who have only been accustomed to the analysis of arsenious acid in a pure and unmixed state. We must refer our readers to the work itself for a description of it. (p. 28.) Thus then here no metallic stains are collected for analysis; indeed the production of them at all only becomes a collateral part of the process; and the result would not be the less satisfactory, although this part of the analysis were altogether omitted. In short the experimentalist has nothing to do with the production of metallic stains; he has merely to determine the chemical characters of arsenious acid, and there are, we believe, very few of the modern race of practitioners who are not fully competent to this.

"The results which we have obtained in experimenting on the remains of poisoned animals by the process just described have been so exact, that in any case of poisoning, we do not hesitate to say, less than two ounces of animal matter (50 grammes de chair) will yield a ponderable quantity of poison, either in the state of arsenious acid, of metal, or in any other form of arsenical combination, according to the pleasure of the operator. Henceforth, it will not therefore be necessary to find traces of arsenic in a body, or to examine chemically the whole of a subject. In the greater number of cases, about two pounds of the viscera or of the muscles will suffice. A trial of a few drachms will serve to give sufficient evidence to guide the analyst in the subsequent process." (p. 31.)

It is then proposed that in the exhumation of the dead for judicial purposes, especially where decomposition has advanced, only certain portions of the viscera should be taken. Two or three pounds of an organ or of muscular fibre, might be even carbonized, and sent in a dry pulverulent state to any distance for further examination by experienced analysts. The authors propose that a council should be appointed, to whom all such analyses should be referred, and whose unanimous judgment would be stamped with an authority which would render it impregnable to the attacks of the envious or the ignorant. This is an excellent suggestion, and the plan proposed is one which might be adopted with great advantage in our own country. Simple as the details of this process may appear, it is a grave question, on the answer to which we think there can be but one opinion, whether the search after poison, on a charge of murder, should be entrusted to inexperienced hands. The English law unfortunately presumes that all belonging to the medical profession, however remotely, are competent to undertake such investigations, until the contrary appear. The presumption, however, is here on the wrong side; and until this principle is recognized by the legislature, the law must be administered in darkness and uncertainty. The essence of the defence in a charge of criminal poisoning consists in the ability of the counsel to throw doubt upon the vital part of the evidence, the chemical analysis. By the constitution of a medico-legal board, such as that recommended by MM. Danger and Flandin, no risk could be incurred, there would be at once security for the innocent, and certainty of punishment for the guilty.

The conclusions at which the authors arrive are as follows:

"1. That there is no arsenic in the human body in its normal state. 2. That in the carbonization of animal matter a soluble organic compound, consisting of sulphite and phosphite of ammonia, with some animal substance, is produced; and that this, when introduced into Marsh's apparatus, gives rise to stains, resembling in physical and chemical characters those of arsenic. 3. That Marsh's apparatus is not to be relied on in medico-legal cases, except where we burn the arsenuretted hydrogen, and operate on the products of combustion. 4. That where arsenical poisoning is suspected, the analyst must during life examine the fæces or vomited matters; and after death, if this event have resulted from the direct effect of the poison, he will find it diffused throughout the system, even in those organs which are most remote from the centre of poisonous action." (p. 33.)

Our opinion of this process will be judged of from the remarks already made. One of its great merits is, that it is equally adapted both to ordinary and extraordinary analyses. When arsenic is mixed up with organic matter, solid or liquid, it is often very difficult to make the principal test act, namely, sulphuretted hydrogen gas. If arsenic be indicated by the colour of the froth formed in the liquid, or by the production of a yellow compound, it is either suspended, or the precipitate falls slowly, and is liable to be mixed with a large quantity of organic matter, which effectually prevents a satisfactory reduction of the sesquisulphuret to the metallic state. In all cases of this description, as where arsenic is mixed with gruel, soup, coffee, or such like substances, it would be advisable to evaporate the liquid so as to form a dry extract, and then carbonize it in the manner directed with pure sulphuric acid. In this way, within an hour or two an analysis of the most complex liquid might, according to the author, be made, while by the ordinary method the boiling and filtration would occupy several days. It may be further remarked, that if we have already begun the analysis with sulphuretted hydrogen, and converted the arsenic partially to the state of sulphuret, this would not interfere with the application of the process of MM. Danger and Flandin. Should the sesquisulphuret be precipitated in an impure state, it may still be transformed to arsenic acid by this method, and the analysis completed in the usual way.

In the process as applied to the organic tissues, we cannot conceive that it would ever be necessary to determine by analysis the quantity of poison present in a given weight of animal matter. It is not easy to understand under what circumstances a question of this kind would arise. The quantity might, and possibly does vary, according to the length of time which the individual survives. The mere discovery of the poison in any of the tissues of the body would, we imagine, suffice to establish the fact of poisoning. In a case of this description, it could not be urged that the poison might have been designedly introduced after death; its detection in the substance of the liver, spleen, or muscles, would at once set at rest any plausible objections of this kind. The only possible objection might be, that arsenic naturally existed in the body; but this we think is an inadmissible assumption, not to say that it is directly negatived by many experimental trials made on the various organs of healthy subjects.

The report of the committee is on the whole favorable to the process of MM. Danger and Flandin, though they prefer "Orfila's modification of Marsh's apparatus, which in their view admits of our obtaining an equally certain and more speedy result." (Report, p. 27.) The experiments which the authors performed before them, gave very clear and satisfactory results.

They object, however, to that statement of the authors', wherein it is asserted that stains resembling those of arsenic could be produced from certain saline products, resulting from the imperfect carbonization of organic matter. "The viscera of healthy animals were carbonized by the nitric and sulphuric acids and the nitrate of potash separately;" but under these very different circumstances, no result was obtained in the use of Marsh's apparatus, which could create any doubt. "The gas when inflamed yielded nothing but water." (Report, p. 22.) They admit it to be possible that the liquids procured from imperfectly carbonized products, where sulphuric acid is used, might, when placed in Marsh's apparatus, give rise to spots slightly resembling those of arsenic in physical, but differing entirely in chemical characters. Indeed there could be no fear of a mistake upon the point, if the operator had the least experience on the subject. It seems by the report, MM. Danger and Flandin were singularly unfortunate in not being able to produce artificially any of these ambiguous stains before the committee. (Report, p. 23.)

Thus then the source of fallacy which they announced does not appear to exist. It seems indeed, that where the carbonization has been complete, the presence of such salts as the sulphite and phosphite of ammonia would be impossible. If by any chance, stains resembling those of arsenic were produced, they would, according to the committee, be easily distinguished by the negative results obtained with the arsenical tests. This, however, we must observe is quite a subordinate point in the enquiry, since the authors do not profess to rely upon the application of tests to a stain for the evidence of the presence of arsenic. They prefer converting it into a compound, the tests applied to which cannot be objected to by scientific men.

It rather surprises us that all the French chemists seem to be quite ignorant of the very simple method devised by Mr. Marsh about six months since, for determining whether the gas produced were arsenuretted hydrogen or not. The knowledge of it would have spared a vast deal of controversy on the subject of stains, and would have rendered it unnecessary to apply any tests to them to determine their real nature. The method to which we allude is the following: Ignite the gas, and hold, at about one inch above the level of the flame, a piece of porcelain or glass, moistened with ammonio-nitrate of silver. If there be arsenic united to the hydrogen, yellow arsenite of silver is immediately produced; if not, there is no yellow precipitate, but the salt is either blackened or undergoes no change. After this it is absurd to talk of applying chemical reagents to the stains. Paper moistened with hydrosulphuret of ammonia might be substituted, and acetic acid then added to detect sesquisulphuret of arsenic.

There is a far more material point in which the researches of the authors are fully corroborated by the results of the experiments performed before the committee, both by themselves and M. Orfila, namely, that in the various trials made with the most minute precautions, no trace of arsenic could be discovered as a constituent of bone, or of any of the other organs of the human body. (p. 89.)

It appears from their experiments, that Marsh's apparatus will easily detect the presence of arsenic, "when the liquid examined contains only $\frac{1}{1000,000}$th part. Indeed stains begin to be formed when the liquid contains a proportion of about $\frac{1}{2000,000}$th. The stains are not produced better with a large than a small quantity of liquid employed in the apparatus, the proportion of arsenic being the same in the two cases. But they are formed for a longer period in the former than in the latter case. Hence it is always preferable to concentrate the arsenical liquids, and to experiment on a small bulk: the stains thus procured are much more perfect." (p. 83.)

We have lately had occasion to examine the stomach and duodenum of a female who died in about thirty-six hours from the acute form of arsenical poisoning. The viscera had been preserved for about five weeks in spirit. We, in this case, adopted the process recommended by MM. Danger and Flandin.

About two ounces of the viscera were divided into small pieces and carbonized by sulphuric acid. So much froth was formed as to render it necessary to watch the decomposition closely. It was found to be a matter of some difficulty to expel the whole of the sulphuric acid from the carbonaceous residue; and although quite dry when first removed it speedily reabsorbed water from the acid present in it. The same difficulty occurred with the nitro-muriatic acid; and on adding to the aqueous solution, nitrate of silver, and ammonia, a yellow precipitate fell down closely resembling arsenite of silver. This was owing to the presence of a phosphate dissolved by the traces of acid, for of course no arsenious acid could be present. We consider, however, that a result of this kind, unless pointed out, might lead to fallacy. No trace of arsenic was detected in the solution.

It might be said that the acids would be easily expelled by the application of a higher temperature; but we have already remarked that we think there is some risk in using so high a temperature in the carbonization as that recommended by MM. Danger and Flandin. It is difficult to understand chemically how arsenic or any of its compounds can be heated to 700° in contact with carbon without being entirely reduced and volatilized. We have thought it right to make these remarks, since, however satisfactory the process may be in the hands of the authors, it is certain that some practice is necessary for success; and it is also possible that arsenic may not be always detected in so small a quantity of organic matter as two or three ounces.

We now come to the Report of the Academy on the various modifications of Marsh's apparatus, proposed by different chemists. As many of these must be entirely new to English readers, we shall briefly notice them, more especially since, in cases where circumstances prevent the employment of one plan, the experimentalist may easily select another.

M. Lassaigne has proposed to pass the arsenuretted hydrogen gas into a solution of nitrate of silver. Metallic silver is, according to him, precipitated, and the solution contains arsenious acid. When it is supposed that all the arsenic is evolved, the surplus nitrate of silver may be thrown down by muriatic acid, and we have a solution which, when evaporated, yields only arsenious acid. (p. 57.)

The committee satisfied themselves that arsenic might thus be separated; but the presence of that substance in a liquid could not be inferred merely from the decomposition of the solution of nitrate on passing into it the hydrogen gas. Sulphur or carbon contained in zinc might give rise to a similar change, and where the operation was carried on in a vessel exposed to light, pure hydrogen itself might decompose the nitrate. Hence arsenic cannot be admitted to exist in a liquid evolving hydrogen, except in those cases where it is actually separated from the solution after the decomposition. It has been suggested that a solution of chlorine or of an alkaline chloride might equally answer the purpose.

We feel it due to Dr. Clark, of Aberdeen, to state that a month before M. Lassaigne had deposited his memoir with the Academy of Sciences, Dr. Clark had read a paper on this mode of detecting minute portions of arsenic, in 1840, at the meeting of the British Association at Glasgow. By means of the nitrate of silver thus employed, Dr. Clark had detected arsenic in most commercial specimens of tin and zinc. He passed the gas in the first instance through a solution of nitrate of lead, which served to detect and separate sulphur, while it had no effect on arsenuretted hydrogen. According to Dr. Clark, the precipitate formed in a solution of nitrate of silver is arsenuret of silver, and not metallic silver, as stated by Lassaigne. Since this gentleman has been able to procure arsenic from the precipitate, it is clear that Lassaigne's theory cannot be correct; and that if we trust solely to the analysis of the residuary liquid, as recommended by the French chemist, there may be little or no arsenic present. Dr. Clark has also announced a fact, of which M. Lassaigne does not seem to be aware, namely, that antimony contained in hydrogen produces the same effect on nitrate of silver as arsenic; hence, before a process of this kind can be safely employed, it is necessary that further experiments should be made.

In a letter addressed to the Academy by M. Signoret, it was stated that metallic stains might be procured with Marsh's apparatus, from a liquid containing only $\frac{1}{200,000,000}$th of arsenious acid. Many experiments were made by him on different specimens of zinc and sulphuric acid procured from different establishments, and he found that they all yielded traces of arsenic. His conclusion is, that it is impossible to obtain pure specimens of these substances in commerce, a statement, if true, of great importance to medical jurists. (p. 58.)

If this assertion of M. Signoret be correct, this will completely overthrow the process of MM. Danger and Flandin for the carbonization of organic matter, and the extraction of arsenic from the human body. We think it therefore right to state their reply, with the conclusions drawn by the committee on this very subject from the experiments performed before them. Commercial zinc may be easily procured free from arsenic; that which has been laminated is the most pure, and should be selected for these experiments. Orfila, however, in many experiments found very few specimens that contained arsenic. Danger and Flandin dissolved slowly in diluted sulphuric acid one pound and a half of laminated zinc, and carefully examined the hydrogen evolved; it did not yield a trace of arsenic, and the zinc was completely dissolved. (p. 77.) In other experiments the results were equally negative. Orfila in a similar way operated on *four pounds* of granulated zinc at one time; but not a trace of arsenic was procured. We believe the presence of arsenic in zinc to be the exception to the rule. In many experiments for several years with *laminated* zinc, we have never found any; and we think with the committee, that this is the state in which the metal zinc should be employed by medical jurists.

With regard to sulphuric acid it has been lately shown by Dr. Rees that this substance is sometimes impregnated with a large quantity of arsenic; probably where it is made from the sulphur obtained from iron pyrites. It is, however, easy to detect this impurity, and to avoid the use of an acid thus contaminated. Before proceeding to carbonize organic matter, or to employ Marsh's test for the saline residue, it is always necessary to determine the purity of the sulphuric acid and zinc; and MM. Danger and Flandin very properly recommend, that we should try these substances in the same quantities as those actually employed in the process. (p. 88.)

We think, therefore, that when common care is taken, the objections of M. Signoret can have no practical force.

M. Coulier states in his letter to the Academy, that stains resembling those of arsenic may be produced on some kinds of glass or porcelain, where the hydrogen is absolutely pure and free from that metal. This is ascribed to the oxide of lead in the glass or glaze being reduced by the gas. We consider this an absurd objection : out of many hundreds of experiments, we have never seen any result of the kind. The glass or porcelain breaks before any such reducing power is exerted by the hydrogen.

The last plan noticed is that of MM. Köppelin and Kampmann. In this case the arsenuretted hydrogen is made to pass through a horizontal tube filled with the chloride of calcium, so that it is thoroughly dried. Heat is then applied to one part of the tube; and as the gas is easily decomposed at a high temperature, hydrogen passes away, while the metal is deposited in a bright metallic ring or sublimate, at a short distance from the heated spot. Other metals held dissolved by hydrogen are deposited in that portion of the tube to which the heat is directly applied; they are not carried forward like arsenic. The ring of metal may be easily examined, and its arsenical characters determined by the usual reagents. The undecomposed portion of arsenuretted hydrogen may either be burnt as it escapes from the open end of the tube, or may be made to pass into a solution of nitrate of silver, in which case it will be entirely condensed and decomposed. Thus, in this way, every trace of arsenic from the poisoned material may be procured.

There are many advantages about this last process; but we do not see the necessity for drying the gas. A slight inclination of the tube towards the generating vessel, would answer all the purpose of removing any portions of liquid that might be carried over. This gas being highly poisonous, it would be always advisable (where there is no stop-cock to command its escape,) to allow any undecomposed portions to pass into a solution of nitrate of silver. This will guard the operator from risk, and will prevent the loss of any portion of arsenic.

We thus conclude our notice of the work of MM. Danger and Flandin. The matter is both original and good, but we must condemn the total want of arrangement displayed in it. The subject is treated in a most confused manner : their own process is described three times, and their statements are carelessly mixed up with those of the committee appointed to report on the value of their researches. We should recommend the work to be reprinted, and more order observed, so that a reader might not have to search over the whole volume for a complete knowledge of the results which they have obtained. The angry discussions at the Academy should be altogether omitted; these occupy half the volume, and, however interesting to the parties concerned, they are quite out of place in a work professedly devoted to science. We must, however, do the authors the justice to state, that in no part do they show a controversial spirit; they claim no merit for themselves; they leave their views to the judgment of the profession, without assuming that they are perfect or free from fallacy.

We shall now proceed to examine Orfila's report of the proceedings of the committee of the Royal Academy of Medicine. This pamphlet has been published by Orfila not only as an indication of his views but for the purpose of refuting certain statements made against them by MM. Magendie and Gerdy. The report which professes to be the same as that referred to by MM. Danger and Flandin, differs from that published in their work, in regard to the conclusions drawn by the committee. The differences, however, are not very material, and do not affect substantially the merits of the processes pursued by the two parties.

It will only be necessary for us here to advert to the modification which M. Orfila has made in his processes since our review of his Memoirs on Poisoning. We there examined his views of the characters of arsenical and spurious stains. The committee, as we have seen, have reported that the spurious stains cannot, when produced, be confounded chemically with those resulting from arsenic; but while this is admitted, it may be inferred from their report generally, that it would be better to collect the arsenic in a more tangible form, and test it directly in the usual way. A new kind of apparatus was proposed by M. Orfila to obviate the objection arising from the occasional production of spurious stains. A figure of this is given, for which we must refer to the pamphlet (p. 18); but we may remark it is similar to that of MM. Köppelin and Kampmann, just now described. Instead of using chloride of calcium for drying the gas, M. Orfila introduces a small portion of asbestos into the horizontal tube. This detains any extraneous matter; and when the portion of the tube containing the asbestos is heated, the metallic arsenic is deposited in a distinct ring at a short distance beyond it.

The next material point relates to the process employed by Orfila for the carbonization of animal matter. In our review of his memoirs it was stated, that he gave the preference to the use of nitric acid; but the committee consider that there are many objections to the employment of this agent. In the first place it is requisite to use it in very large quantities, much is lost by evaporation before the animal matter is attacked, there is a large quantity of froth produced, and by this as well as by accidental combustion, which occasionally takes place, the greater portion of the arsenic is dissipated. They allow that arsenic may be in this way detected; but the carbonizing process succeeds better with sulphuric acid or nitrate of potash.

The use of nitre for detecting arsenic in poisoned subjects by incineration, was suggested by Rapp thirty years ago, and it has been used for this purpose by many English and continental chemists since that time. It is not necessary for us to describe Rapp's process; since it has been much improved by Orfila. Orfila's plan, which is considered by the academy to be superior, as a means of carbonization, to that of MM. Danger and Flandin, is very simple. If it be a suspected liquid, we dissolve nitre in it, and evaporate to dryness, so as to ensure a perfect mixture of the salt with the organic matter. If it be a soft organ, as the liver, it is to be bruised and mixed into a paste in a mortar, with nearly double its weight of nitre.* The mass is then dried in a porcelain capsule at a gentle heat. When thoroughly dry, it is projected by small portions into a Hessian crucible, made red hot. The calcined residue ought to be perfectly white: should this not be the case, more nitre must

* We have not found this process of mixing so easy as it is represented to be, when we are operating on the stomach and intestines.

be added, and a fresh calcination made. When cold the saline residue is dissolved out of the crucible by a small quantity of water. If much nitre has been employed a large quantity of water will be employed: the fused nitre adheres closely to the crucible. Concentrated sulphuric acid is added until there is no longer effervescence; the whole of the nitric acid is afterwards expelled by continued boiling; the solution which contains sulphate and arseniate of potash, if arsenic were present, is then introduced into Marsh's apparatus. (p. 32.)

This process is approved of by the committee owing to the very satisfactory nature of the results, but they admit that there are certainly cases in which it would be better to employ sulphuric acid as the carbonizing agent. Thus where the matters analysed are of a fatty nature, combustion might take place with so violent a flame, as to occasion a loss of arsenic. In respect to delicacy the processes are nearly equal, and a good result may be obtained by both. In some instances, the product in arsenic was greater with nitre than with sulphuric acid (p. 34). There is great embarrassment in having a large quantity of sulphate of potash mixed up with the arseniate; and we really do not see why, with some modifications, sulphuric acid might not supersede nitre as the agent. It has the advantage of being a more convenient and speedy means of decomposing organic matter, and the arsenic is ultimately obtained in a pure state. It may be a question, however, whether nitre does not more effectually fix the arsenic in the saline state than sulphuric acid. According to the report of the committee, there seems to be but little difference in this respect.

Orfila seems quite satisfied that the committee have reported that he was the first chemist who detected arsenic in the substance of the animal organs, and that the urinary secretion in animals poisoned by arsenic, is not always suspended, (p. 36;) but we are sorry to see that he wishes to strip MM. Danger and Flandin of all credit for their ingenious process, by assigning the merit of it to another, in our opinion without sufficient grounds (p. 41). He accuses them of inconceivable presumption, condemns their apparatus as complicated, and says what does not appear to us to be the fact, that the committee did not recommend its employment (p. 42). Had this been the case, the authors would hardly have received the thanks of the Academy (p. 37).

There is another point in this memoir which requires especial notice, since Orfila passes it over in a few words; it is that which refers to the presence of arsenic in the bones of the human body. In our former review, we took occasion to denounce this as a startling novelty, and a statement not to be admitted upon the very weak evidence upon which it was based. The analysis of bone by many chemists had not led to the confirmation of Orfila's assertion, but rather tended to negative it. Since our former remarks, Dr. Rees has carefully examined bone in various experiments, the results of which are inserted in a late number of the Guy's Reports. They were such as all chemists might have anticipated, not a trace of arsenic could be discovered. We have already seen from the report made by the committee of the Academy, on the experiments performed before them by MM. Danger and Flandin, that no arsenic could be detected either in bone or any of the organs of the body, of a person who had not taken poison. M. Andonard, a chemist of Beziers, had also examined bone without finding arsenic. Orfila remarks in a note to the Report; "How is such a contradiction to be explained? In 1839 we obtained from bone stains which were truly arsenical, and which possessed *all the physical and chemical characters* of those produced by arsenic. These results were constant. But in an exact repetition of these processes (before the committee), and employing reagents as pure as those formerly used, we could not extract any arsenic. There is some obscurity here which requires to be cleared up." (p. 42.)

Orfila affirms that the committee have not denied the *existence* of arsenic as a constituent of bone, they have merely said it was not produced before them. We will give him the benefit of this admission; but in these medico-legal matters, as in those which are purely legal, we would say, "De non existentibus et non apparentibus, eadem est ratio." It would not, we think, be difficult to clear up the obscurity which hangs over this subject, or to account for these discrepant results. We believe that the stains observed by Orfila in examining bone were either those spurious stains which all must have met with in using Marsh's apparatus, or if they really possessed the chemical characters of arsenic, then the arsenic must have been unknowingly introduced during the process, from some residuary portion in the tube, or from impurity in the materials employed. That one or the other of these explanations must be correct appears probable from the fact, that many chemists who have tried Orfila's experiments have not met with like results; and that he himself, in repeating them before the committee, completely failed in reproducing them. It is clear from this that chemical tests cannot be safely relied on when applied to such minute portions of matter as must have existed in the stains said to have been procured from bone by Orfila.

After this report, what becomes of the arsenic which is stated to exist in the soil of most cemeteries—and the origin of which was ascribed to decomposed bone? MM. Danger and Flandin have shown that stains thus procured were not really of an arsenical nature; and certainly the explanation of the origin of the supposed arsenic falls to the ground. These announcements of Orfila tended at the time to shake the foundation of all medical evidence in cases of arsenical poisoning; and we must congratulate medical jurists upon their having been clearly proved to be erroneous. We had anticipated this result in our former remarks on Orfila's memoirs. This fact shows, perhaps as strikingly as any other we can adduce, the dangerous inferences into which an experienced man may be led by relying too strongly on tests applied to minute stains produced from unknown substances.

We can hardly look upon this report as a separate work of Orfila's: it is rather a defence of his opinions against the many attacks made upon them. Some of these attacks were trivial and absurd, and Orfila would have acted wisely in leaving them unnoticed. Other objections to his views, involving matters of high practical importance, he has not discussed with that calmness which we should have expected from a man of his experience and sagacity. It is no disgrace to a deservedly celebrated man to have fallen into an error; but it is to be regretted that he should be disposed to persist in it even against evidence, which, on other subjects in which he was less personally concerned, he would not hesitate to admit. The reputation of Orfila will live as long as legal medicine shall be known or studied; and it would be to us a matter of regret if our remarks should be supposed to weigh heavily on a man who has by his indefatigable perseverance and extensive knowledge earned so noble a reputation in medico-legal science. Our observations have been directed not against him, but against opinions which, it seemed to us, might if unnoticed, lead to pernicious consequences.

We do not consider it necessary to lengthen this article by any additional remarks on the last two works in the list. The Memoir on Poisoning extracted from the Memoirs of the Royal Academy of Medicine, gives a short history of the several cases of poisoning by arsenic, in which the evidence of Orfila has been received and acted on by courts of law, in relation to the presence of arsenic in the organic tissues of the body. Among these is the well-known case of Madame Laffarge. They will be perused with some interest by the medical jurist, more especially, since this kind of evidence of the presence of poison in the organs of the dead, has never yet been offered to an English court of law. It is unnecessary to refer to the plans adopted by Orfila to detect poison in these cases, since he has now in some measure laid them aside; and we have already noticed in this article his improvements to a much later date than that to which these cases refer.

The work of M. Beaufort is a mere reprint of the different memoirs of Orfila on the subject of arsenical poisoning. A large portion of the work is occupied by the report of the Academy, already noticed. We find nothing new in the shape of comment or experiment; and it would be impossible to make an extract from any portion of it without recapitulating matters already fully examined in this and our former review. To those who wish to have a compilation of Orfila's labours in a small space, we can recommend this work of Dr. Beaufort's. The compiler is, however, evidently a partisan; and his opinions of the relative merits of the process of Orfila and others, must therefore be taken with some limitation.

---

